# Short-Chain Fatty Acids Improve Hippocampal Atrophy, Ventricular Dilatation and Cognitive Function Decline in Aged Mice

**DOI:** 10.14336/AD.2025.0426

**Published:** 2026-05-26

**Authors:** Pei-Ju Lee, Yu-Chun Lo, You-Yin Chen, Chaur-Jong Hu, Yen-Kuang Lin, Quoc Thao Trang Pham, Nicholas Keyi Sim, Chee Kin Then, Shing-Chuan Shen

**Affiliations:** ^1^Graduate Institute of Medical Sciences, College of Medicine, Taipei Medical University, Taipei, Taiwan.; ^2^Department of Oncology, University of Oxford, Oxford, UK.; ^3^Ph.D. Program in Medical Neuroscience, College of Medical Science and Technology, Taipei Medical University, Taipei, Taiwan.; ^4^Department of Biomedical Engineering, National Yang Ming Chiao Tung University, Taipei, Taiwan.; ^5^Department of Neurology, Shuang Ho Hospital, College of Medicine, Taipei Medical University, New Taipei, Taiwan.; ^6^Graduate Institute of Clinical Medicine, College of Medicine, Taipei Medical University, Taipei, Taiwan.; ^7^Graduate Institute of Athletics and Coaching Science, National Taiwan Sport University, Taoyuan, Taiwan.; ^8^International Ph.D. Program in Cell Therapy and Regenerative Medicine, College of Medicine, Taipei Medical University, Taipei, Taiwan.; ^9^Centre for Neurosciences, Norfolk and Norwich University Hospitals NHS Foundation Trust, Norwich, UK.; ^10^Department of Radiation Oncology, Shuang Ho Hospital, Taipei Medical University, New Taipei City, Taiwan.; ^11^Core Laboratory of Human Microbiome, Office of Research and Development, Taipei Medical University, Taipei, Taiwan.; ^12^International Master/Ph.D. Program in Medicine, College of Medicine, Taipei Medical University, Taipei, Taiwan.; ^13^Department of Dermatology, School of Medicine, College of Medicine, Taipei Medical University, Taipei, Taiwan.

**Keywords:** Short-chain fatty acid, gut microbiota, hippocampal atrophy

## Abstract

Many countries are becoming aged or super-aged societies. This demographic shift causes substantial social, economic, and personal costs directly and indirectly attributable to cognitive decline. Recent studies have shown that dietary fiber can slow down memory decline, with short-chain fatty acids being the primary metabolites produced by gut microbiota from the fermentation of dietary fiber. Despite this, there are limited studies investigating the effect of SCFAs on age-related cognitive function and morphological changes of the brain. In this study, we used B6C3F1 male mice at the age of 3 months and treated them with water, low dose, and high dose SCFAs for 9 months. We assessed their short- and long-term cognitive functions using the Novel Object Recognition test, Morris Water Maze, and Rotarod test. Their brain structure was assessed by 7 Tetra Magnetic Resonance Imaging (7TMRI) and gut microbiota analyzed by 16S rRNA sequencing. Our results show that both short-term and long-term SCFA treatment significantly improve cognitive deficits in the Novel Objective Recognition test and Morris Water Maze tests. Additionally, the 7T MRI results show that SCFAs mitigated hippocampal atrophy compared to the control group. These improvements were accompanied by alteration of gut microbiota composition. We also found that, after treatment, the beneficial gut microbiota *Alloprevotella* was positively correlated with hippocampal volume. Therefore, we propose that SCFAs may be a promising therapeutic strategy to counteract age-related cognitive decline.

## INTRODUCTION

The human lifespan has been significantly prolonged by modern medicine and technology. In Western countries, life expectancy has surged by approximately 20 years since 1950 [1], and projections suggest that by 2050, about 10% of individuals in developed countries will live beyond 80 years of age [2]. However, a notable gap remains between life expectancy and healthy life expectancy, indicating that many people spend a significant portion of their later years living with chronic diseases and functional limitations [3]. Among these, age-related cognitive decline is particularly concerning, posing a major burden not only on affected individuals and caregivers but also on healthcare systems and the broader economy. Despite growing awareness, effective interventions to delay cognitive aging remain limited, with most focused-on lifestyle modifications such as physical activity and dietary strategies.

Cognitive function encompasses key mental abilities including memory, attention, learning and adapting to new situations. As the brain ages, structural and functional deterioration leads to decline in memory, processing speed, and attention span [4].Brain volume has been shown to decrease by about 5% per decade after age 40 [5]. In animal models of aging, ventricular enlargement and hippocampal atrophy are well documented [6]. Additionally, degeneration of long-range myelinated fibers impairs communication between brain regions, contributing to cognitive deficits in elderly [7].

Lifestyle interventions such as aerobic exercise and dietary changes have shown promise in promoting healthy brain aging [8, 9]. Regular aerobic activity enhances neuroplasticity and memory by stimulating the production of brain-derived neurotrophic factor (BDNF) and supporting hippocampal function in both animal and human studies [10, 11]. Similarly, several dietary approaches have been associated with cognitive benefits in aging [12]. The Mediterranean diet has been linked to improved memory and cognition in individuals with mild cognitive impairment (MCI) [13], slower memory decline [14], and reduced risk of Alzheimer's disease (AD) [15, 16].

A key component of the Mediterranean diet is dietary fiber, which is fermented by gut microbiota into short-chain fatty acids (SCFAs), namely acetate, propionate, and butyrate [17, 18]. These microbial metabolites play critical roles in maintaining gut epithelial health, supporting intestinal barrier integrity, and regulating immune responses [18, 19]. Importantly, SCFAs can cross the blood-brain barrier (BBB) and have been shown to promote BBB integrity and enhance synaptic plasticity [20–22]. In animal models, SCFAs have demonstrated beneficial effects in mitigating aging-related symptoms, such as spatial memory decline in the CK-p25 mouse model [23, 24]. Butyrate has been shown to improve cognitive performance in models of vascular dementia and high-fat diet induced cognitive impairment [25]. It also increases brain-derived neurotrophic factor (BDNF) levels in models of pneumonia-induced meningitis [26, 27]. Together, these findings suggest that SCFAs may help reverse spatial memory deficits and reduce hippocampal damage in aging and neurodegeneration.

These results underscore the significance of the gut-brain axis, a bidirectional communication system involving neural, immune, metabolic, and endocrine pathways that link the gastrointestinal tract and the central nervous system [28–30]. Consequently, dietary interventions aimed at modulating gut microbiota have emerged as promising strategies to support cognitive health. Gut microbiota influence brain function through a wide range of metabolites [31]. For instance, supplementation with *Clostridium butyricum* increases butyrate levels in the brain [32], while metabolites produced by *Lactobacillus*, such as SCFAs, serotonin, and acetylcholine, have been shown to enhance memory and sleep quality in mice [33, 34]. Tryptophan metabolites produced by *Bacillus* species also improve cognitive function in pig models [35–37].

In addition, dietary polyphenols such as those found in pomegranate and resveratrol-rich food have been found to modulate the microbiome, reduce inflammation, and promote neuroprotection through increased SCFA production. Resveratrol has been shown to lower the abundance of pathogenic bacteria, oxidative stress, and inflammation in cardiovascular disease models [38, 39]. Similarly, polyphenols in pomegranate have been found to increase the abundance of SCFA-producing bacteria, which may contribute to their anti-inflammatory and neuroprotective effects [40, 41]. SCFAs, therefore, play a central role in shaping the gut microbial landscape and influencing host physiology. Nonetheless, the direct effects of SCFA treatment on cognitive function, particular in aging, remain inadequately understood.

Increasing attention has also been paid to the role of inflammation in cognitive aging. Chronic inflammation is a hallmark of aging and is linked to neurodegeneration [42]. Toll-like receptors (TLRs), especially TLR4, are key components of the innate immune system and play a pivotal role in recognizing microbial signals. TLR4 activation can promote microglial activation and stimulate the release of pro-inflammatory cytokines, thereby contributing to age-related neuroinflammation and cognitive decline [43]. Aging rats commonly exhibit memory impairment, which has been associated with increased intestinal epithelial permeability resulting from gut microbiota dysbiosis. This breakdown of the gut barrier is thought to trigger systemic inflammation and activate the TLR4/NF-κB signaling pathway within the hippocampus, contributing to cognitive decline [44]. Despite these promising insights, the direct effects of SCFA treatment on brain structure and cognitive function in the context of aging remain underexplored. The specific mechanisms by which SCFAs interact with gut microbiota, inflammation, and the central nervous system also require further investigation. Our findings indicate a potential link between SCFA treatment and TLR4-related immune modulation; however further research is required to clarify this relationship and determine its relevance to aging.

In this study, we investigate the impacts of SCFA treatment on cognitive function, brain structural changes, and gut microbiota composition in aged mice. We also explore potential links between SCFA treatment and inflammatory signaling. Our findings contribute to a growing body of evidence suggesting that gut microbiota and their metabolites play a key role in age-related cognitive decline and point to SCFAs and dietary fiber as promising therapeutic targets for promoting healthy brain aging.

## MATERIALS AND METHODS

### Animal and treatment

All experiments were approved by the Institute Animal Care and Use Committee (IACUC) of Taipei Medical University, Taiwan. B6C3F1 mice were purchased from BioLASCO (Charles River Licensee Corp, Yi-Lan, Taiwan). Only male mice were used in this study to avoid the potential confounding variable of the mouse estrous cycle. The mice were provided with food and water, and they were housed under controlled conditions of a 12-hour light/dark cycle, along with temperature and humidity regulation. Daily inspections were conducted, and body weights were recorded weekly to monitor their overall health. In this study, mice were randomly assigned to three groups using a computer-generated randomization sequence: control (n=8), low-dose SCFAs (n=9), and high-dose SCFAs (n=8). The group sizes were determined based on a priori power analysis using G*Power 3.1, which ensured that the sample size would be sufficient to detect meaningful differences with appropriate statistical power, while also aligning with established guidelines for behavioral experiments in rodents and ethical principles for animal research. The SCFAs used in this study were administered in powder form, including sodium acetate (Sigma-Aldrich, Cat# S7545), sodium propionate (Sigma-Aldrich, Cat# P1880), and sodium butyrate (Sigma-Aldrich, Cat# 303410). The compounds were dissolved in the drinking water and supplied at two concentration levels: low doses (33.75mM sodium acetate, 12.95mM sodium propionate, 20mM sodium butyrate) and high doses (67.5mM sodium acetate, 25.9 mM sodium propionate, 40mM sodium butyrate). The drinking water was refreshed twice a week. The high-dose regimen was selected based on prior literature demonstrating both its physiological relevance, safety profile, and its ability to influence behavior in vivo.

### Novel object recognition test

To evaluate the recognition memory of the mice, we conducted a novel object recognition test at three time points, before treatment, 6 months and 9 months after commencing SCFA treatment. NORT depends on the exploration of the novel object habit of mice. We placed the mice into the white square chamber (around 45 cm x 45 cm x 45 cm). The test spanned a duration of 4 days, encompassing an initial habituation period lasting 5 minutes on the first day, followed by 20 minutes on the second and third days. All materials used in behavior testing were thoroughly cleaned with 70% ethanol between trials to minimize bias caused by olfactory cues. On testing day, two identical objects, referred to as familiar objects, were placed symmetrically for a duration of 10 minutes, allowing the mice to explore them freely. During the retention trial, lasting 5 minutes, one of the two familiar objects was replaced with a novel object, and the exploration time for each object was recorded. Exploration time was identified when the snout was directed towards the object at a distance ≤ 2mm. Movement was observed through the camera positioned above the chamber and analyzed using EthoVision XT 16 software. The recorded metrics included exploration time, frequency, and velocity of the mice. Recognition index (RI) was calculated using the following formula, RI = Time exploring novel object/(Time exploring novel object + Time exploring familiar object) ×100%

### Morris water maze test

The Morris water maze test (MWM) was used to assess the hippocampal-dependent learning and memory abilities. This involved navigating the mouse through opaque water infused with white non-toxic paint to locate a concealed platform. The behavior test performed in a circular tank with a diameter of 120 cm containing water maintained in 25~26 °C. The escape platform in a size of 15 × 15 cm was placed 1 cm below the surface of the water. The maze was virtually divided into four arbitrary, equally spaced quadrants delineated by the cardinal points north, east, south, and west. Visual cues in black on a white background were positioned on the inner wall of the tank. Each daily trial comprised three swimming sessions, during which mice were positioned in the tank facing the wall and initiated from various starting points in a randomized order. The mice had 60 seconds to swim to the platform. Following each trial, the mice were retrieved and returned to their respective home cages. To mitigate the risk of hypothermia, mice were carefully dried with a paper towel after each trial. Monitoring was conducted through a camera positioned directly above the tank, and data analysis was performed using EthoVision XT 16 software. Key metrics such as latency to the platform, distance travel, and mean swimming speed were measured to assess spatial memory.

### Rotarod test

To evaluate muscle endurance and motor learning ability, we used a 5-lane Rota-Rod instrument designed for mice (Orchid RR02, GERIN Technology Co., Ltd). The mice were placed on the rotarod, initiating at a rotation rate of 4 rpm. The rotarod underwent uniform acceleration, reaching 40 rpm over a 5-minute period, with trials concluding when the mice fell from the rod. The duration of time until the mice fell, the speed of rotation, and the distance travel were recorded for analysis.

### Magnetic Resonance Imaging (7T MRI)

To examine the structural changes in the brain, mice underwent Magnetic Resonance Imaging after 9 months of SCFA treatment. The imaging procedure took place on a 7T small animal scanner (Pharma Scan 70/16 US; 16-cm inner bore diameter) at the animal center of Taipei Medical University, Taipei, Taiwan. T2-weighted images were acquired using a fast spin-echo pulse sequence with two effective echo times (50 and 80 milliseconds), TR=2 seconds, a 90° flip-over angle, Percent Phase Field of View: 100, FOV=2.56 x 2.56 cm, matrix=128 x 128, Repetition Time: 2500 milliseconds, echo time: 33 milliseconds, echo train length 8, and 8 signal averages. The imaging was conducted using the ParaVision 6.0 system. Mice were anesthetized with 2.5% isoflurane, and during scanning, they were maintained under 1–2% isoflurane while their breathing rate was monitored.

To investigate whether different doses of SCFA treatment were associated with alterations in brain volume in mice, we measured the volumes of various brain regions on MRI images, including the hippocampus, CA1, CA2, CA3, dentate gyrus (DG), lateral ventricle, third ventricle, and fourth ventricle. Prior to analysis, MRI images underwent conversion from DICOM to NifTI format using MRIcroGL, involving axis orientation correction, denoising, and orientation correction. The MRI images were initially segmented into 159 structures in each hemisphere using the Multiple Automatically Generated Templates (MAGeT) brain segmentation algorithm. MAGeT generated multiple segmentations for each subject by creating a template library through pairwise nonlinear image registration. The final segmentations for each subject were determined using a majority-vote label fusion method. To ensure label consistency with the underlying anatomy, quality control was performed by visually inspecting the alignment of these final segmentations on each image using AFNI software. For the calculation of the region of interest (ROI), the FSL (FMRIB Software Library) EYES software was employed to segment and create individual masks. The actual size of the target brain region was then computed using Matlab_R2018b (MATrix LABoratory), where pixels were converted to volume measurements (mm^3^).

### 16S rRNA gene sequencing

We collected mouse feces weighing around 0.2 g after 9 months of initiating SCFA treatment and stored them at -80°C. Bacterial genomic DNA extraction was performed using the QIAamp Fast DNA Stool Mini Kit following the manufacturer’s instructions. Fecal samples were resuspended in 180 μl of TE buffer containing lysozyme (final concentration 10 mg/ml). The mixture, combined with glass beads, underwent homogenization for 30 seconds using a FastPrep 24 homogenizer (MP Biomedicals, USA) to ensure complete disruption of cell walls and efficient DNA release. DNA concentrations were measured using a BioDrop spectrophotometer (Biochrom Ltd., Cambridge, UK), and the DNA samples were stored at −20°C for future processing. The V3-V4 region (301 bp) of the 16S rRNA gene was amplified according to the 16S metagenomic sequencing library preparation procedure (Illumina). Raw sequencing data were generated, resulting in a total of 126,879 raw reads. The quality of the sequencing data was assessed using FastQC, Trimmomatic, plotQualityProfile, and filterAndTrim. Quality filtering, denoising, and chimera removal were performed using the DADA2 plugin in QIIME2 (version 2023.2), which retained high-quality reads and resulted in the identification of 583 unique Amplicon Sequence Variants (ASVs). Rarefaction was performed at a depth of 130,001 reads per sample, based on alpha rarefaction curve analysis. Samples with sequencing depths below this threshold were excluded from downstream diversity analysis. Taxonomic assignment was carried out using a Naive Bayes classifier trained on the SILVA 138 database for the V3–V4 region. The Uparse algorithm was applied for operational taxonomic unit (OTU) clustering with a 97% similarity threshold. Alpha-diversity metrics, including Shannon and chao1 index, and beta-diversity were calculated using QIIME 2. The Wilcoxon test was conducted using R (version 2.15.3). Linear discriminant analysis (LDA) and linear discriminant analysis effect size (LEfSe) were employed to identify microbial differences, where p<0.05 indicates statistical significance, and LDA values >4 represented significantly higher abundance.

### Tissue preparations

Following induction of deep anesthesia, mice were euthanized via transcranial perfusion using phosphate-buffered saline (PBS) to eliminate blood from the central nervous system. Whole brains were immediately excised and fixed in 10% neutral-buffered formalin for 24 hours at room temperature. The fixed specimens were then transferred to 70% ethanol and stored at 4 °C until further processing. Brain tissues were carefully trimmed, then dehydrated through a series of increasing ethanol concentrations. After dehydration, samples were cleared in xylene and embedded in paraffin wax. Coronal sections of the brain were cut at a thickness of 4 µm using a microtome. To ensure consistent sampling of the hippocampus, sections were taken approximately 2.3 mm posterior to the bregma, aligning with the anatomical coordinates described in the mouse brain atlas by Paxinos and Franklin (2001), which corresponds to a depth of roughly 8.3 mm from the cranial surface.

### Hematoxyline & Eosin (H&E) staining

For histological evaluation, paraffin-embedded sections were first deparaffinized in xylene and then rehydrated through descending ethanol concentrations. Nuclear staining was carried out using Harris or Mayer’s hematoxylin solution (Sigma-Aldrich, MHS32) for approximately 5 minutes. Excess stains were removed through differentiation in acid alcohol, followed by using 0.1% ammonia water to enhance nuclear contrast. Cytoplasmic structures were then visualized by counterstaining with a 1% eosin Y solution for 1–2 minutes. After staining, the slides were dehydrated through ascending ethanol concentrations (50%, 70%, and 100%), cleared in xylene, and permanently mounted with coverslips. Microscope examination was performed under a bright-field microscope at 10× and 20× magnifications. Tissue sections were analyzed for histopathological features consistent with neurodegeneration, including neuronal cell death and reactive gliosis.

### Immunohistochemistry staining

Paraffin-embedded sections were deparaffinized in xylene and rehydrated through a graded series of ethanol. Antigen retrieval was performed by heating sections in citrate buffer (10mM. pH6.0, ThermoFisher, Cat# 005000) at 95-100°C for 20 minutes using water bath. Endogenous peroxidase activity was blocked by incubating the slides in 3% hydrogen peroxide for 10 minutes. After washing in PBS, the sections were incubated in 5% normal goat serum. The tissues were then incubated overnight in 4°C with a primary antibody (rabbit polyclonal TLR4 antibody, Cat# SKU TA336415, ORGIENE, dilution 1:200). After washing with PBS, sections were incubated with a goat anti-rabbit secondary antibody for 20 minutes at room temperature. Immunoreactivity was visualized using 3,3’ -diaminobenzidine (DAB) as the chromogen, and sections were counterstained lightly with hematoxylin. To quantify TLR4 expressions in the hippocampus, non-overlapping digital images of stained sections were captured at 20× magnification using a Motic EasyScan Pro scanner. Image analysis was performed using ImageJ (NIH). Six regions of interest (ROIs) were defined within the hippocampus using the Polygon selection tool. Images were converted to 8-bit grayscale and thresholded to isolate TLR4-positive cells. The Analyze Particles function was used to count the number of TLR4-positive cells in each ROI. The total number of positive cells was recorded, and the mean number per ROI was calculated. To determine the percentage of TLR4-positive cells, the number of positive cells was divided by the total number of cells in each ROI and multiplied by 100. For statistical analysis, mean TLR4-positive cell counts across experimental groups were compared using one-way ANOVA followed by Tukey’s post hoc test. Statistical significance was defined as *p* < 0.05.

### ELISA

To evaluate inflammation, we collected mouse serum and performed ELISA assays. Flat-bottom 96-well plates were prepared by applying the capture antibody (Mouse IL-6 ELISA MAX™ Deluxe Set, BioLegend, CAT#431304, and TNF-α, ELISA MAX™ Deluxe Set, BioLegend, CAT #430904) in accordance with the supplier's protocol and incubated overnight at 4°C. The following day, nonspecific binding sites were neutralized by introducing 200 μL of 1X Assay Diluent A to each well for 1 hour at ambient temperature. Subsequently, 100 μL of either standard solutions prepared in serially diluted gradient concentrations or pre-diluted mouse serum samples from three groups (control, low-dose, and high-dose) were added to the wells and incubated for 2 hours. Detection antibody, formulated per the manufacturer's recommendation, was added and incubated for an additional hour. A diluted Avidin-HRP conjugate was then introduced for 15 minutes, succeeded by the addition of a 1:1 mixture of chromogenic substrate solutions A and B (TMB substrate). The enzymatic reaction was halted using 2N sulfuric acid after 15 minutes. Between each procedural stage, wells were rinsed three times with PBST (PBS supplemented with 0.05% Tween-20). Optical density was recorded at 450 nm with background correction at 570 nm using an Agilent BioTek Epoch microplate reader.

### Statistical Analysis

Data are presented as mean ± SD. Statistical analyses were performed using GraphPad Prism 9 (GraphPad Software, San Diego, CA). Normality was assessed using the Shapiro-Wilk test. For multiple group comparisons, one-way ANOVA followed by Dunnett’s multiple comparison test was used. For datasets that did not meet the normality assumption, the non-parametric Kruskal-Wallis was applied, followed by Dunnett’s multiple comparison test. Correlations between gut microbiota abundance and hippocampal volume were analyzed using Pearson's correlation coefficient. Statistical significance was defined as p < 0.05.

**Table 1.** General characteristics of the animals.

	Group		
	Control	Low dose	High dose
	n=8	n=9	n=8
**Age (month)**	12	12	12
**Basal body weight (g)**	29.03±3.28	27.84±2.69	29.18±2.39
**Final body weight (g)**	37.22±4.87	34.97±3.52	34.2±3.23
**Water intake (ml/mouse/day)**	8.4±2.65	8.03±2.08	8.08±1.6
**Food intake (g/mouse/day)**	26.71±13.31	26.45±9.23	22.59±8.09

Data are represented as mean ± SD.

## RESULTS

We allocated mice into three groups, namely control (n=8), low-dose SCFAs (n=9) and high-dose SCFAs (n=8), and initiated treatment at 3 months of age and followed up for 9 months. The low-dose SCFA is 33.75 mM acetate, 12.95mM propionate and 20 mM butyrate, while the high-dose SCFA is 67.5 mM acetate, 25.9mM propionate and 40mM butyrate. The average weight, food, and water intake data are presented in Table 1. There were no significant differences observed in body weight (p=0.0788), water intake (p=0.8695), or food intake (p=0.4468) among the three groups.

### Short-term SCFA treatment increased recognition index, spatial memory and learning ability

**Figure 1.**
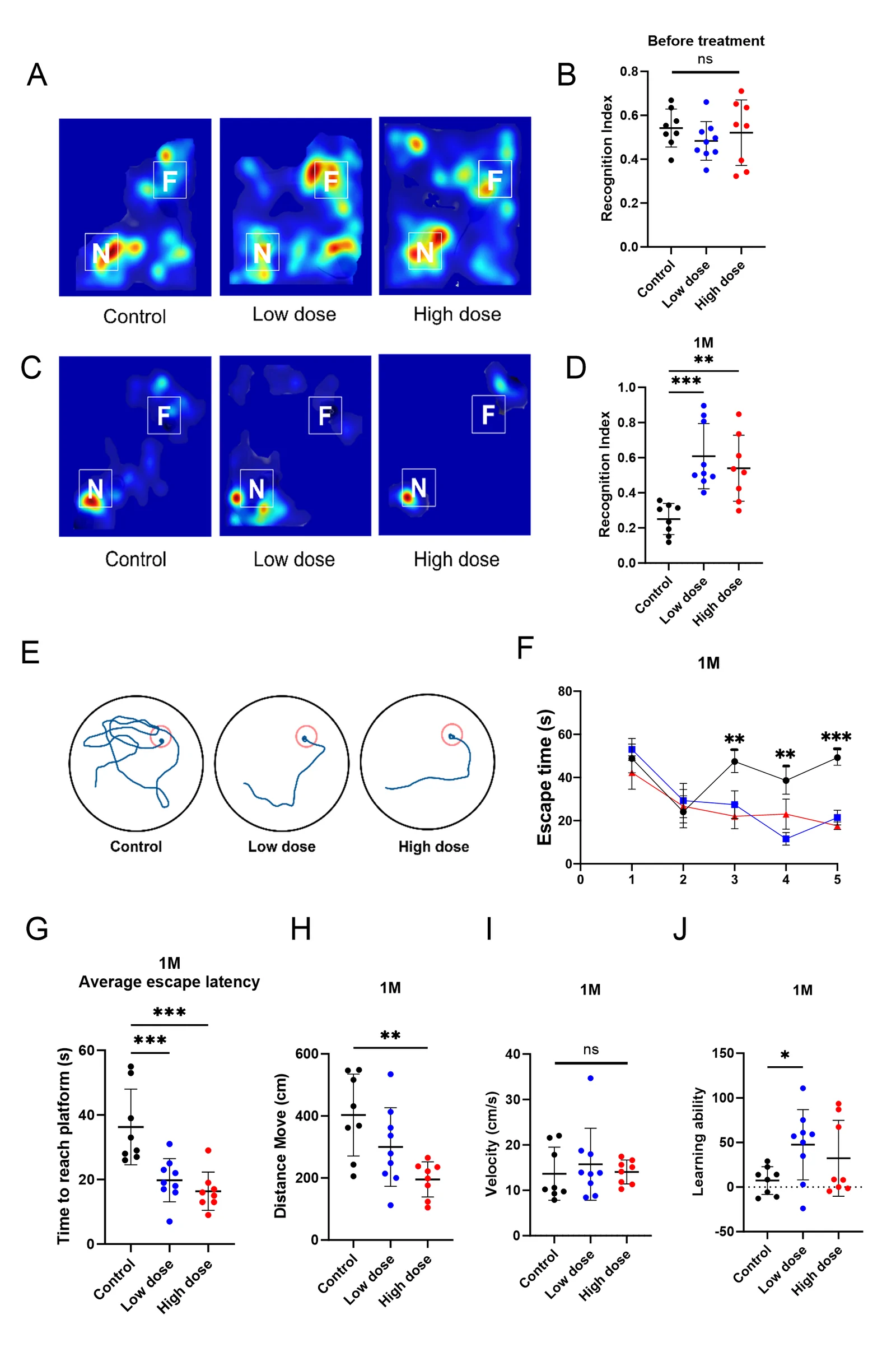
**Short-term SCFA treatment enhances short-term and spatial memory. (A, C)** Novel Object Recognition Test (NORT) and **(E)** Morris Water Maze Test (MWM): Heatmap representative images selected based on the group median value to best reflect central tendency of the data. **(B)** NORT Recognition Index of three groups of mice measured before treatment. **(D)** NORT Recognition Index of mice treated by low-dose and high-dose SCFAs for 1 month vs aged control. **(F)** MWM Escape Time, representing spatial learning ability, recorded during day1 to day5 training. **(E)** MWM Swimming routes, **(G)** mean escape latency, **(H)** distance moved, and **(I)** swimming velocity to the platform were recorded. **(J)** Rotarod Test**:** Motor learning ability was calculated by Trial 9-Trial 1 latency to fall off the rotarod. control, n=8, low-dose, n=9, high-dose, n=8. Normality was assessed using the Shapiro-Wilk test. Statistical differences among groups were analyzed by One-Way ANOVA, followed by Dunnett’s multiple comparison test. For datasets that failed normality, non-parametric Kruskal-Wallis was employed followed by Dunnett’s multiple comparison test. *p<0.05, **p<0.01, ***p<0.001. Data are expressed as mean ± SD.

To assess the baseline cognitive function, the novel object recognition test (NORT) was performed at 3 months of age, prior to SCFA treatment. No significant differences were observed among groups at baseline (p=0.6753, Fig. 1B). One month after SCFA treatment, both the low-dose SCFA (p=0.0062) and high-dose (p=0.0017) SCFA groups showed a significant improvement in recognition index compared to the control group (Fig. 1D), suggesting a beneficial effect of SCFAs on short-term memory. Consistent results were observed in the Morris Water Maze (MWM) test, which assesses spatial learning and memory. Both low-dose (p=0.0006) and high-dose (p=0.0009) SCFA groups exhibited significantly reduced escape latencies compared to controls during days 3 to 5 of the training phase (Fig. 1E and F). Additionally, only the high-dose SCFA group showed a significant reduction in distance traveled to reach the platform (p=0.0056) (Fig. 1H), while swimming speed did not differ among groups (Fig. 1I), indicating that improvements were memory-related rather than due to motor function. Learning ability was further evaluated using the rotarod test, which demonstrated that both low- and high-dose SCFA treatment significantly enhanced motor learning ability (p=0.0494; Fig. 1J). Figure 1 shows representative images of the NORT and MWM tests, selected based on the median value of the relevant quantitative outcome for each group, to best reflect the central tendency of the data (Fig. 1A, C, and E).

### Long-term SCFA treatment slowed aging-related memory decline

**Figure 2.**
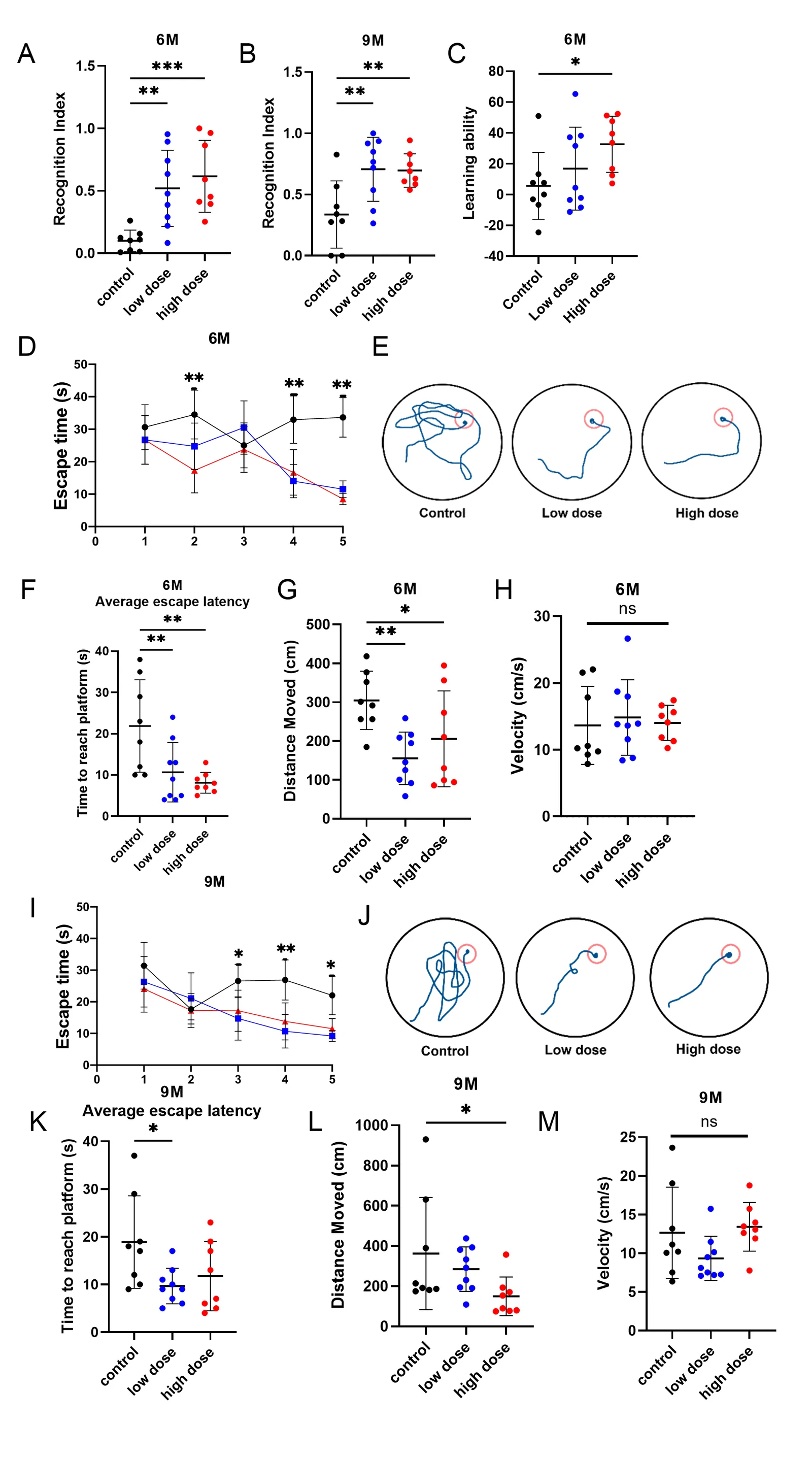
**Long-term SCFA treatment enhances short-term and spatial memory.** The Morris water maze (MWM) Recognition Index of three groups of mice measured after **(A)** 6-month, and **(B)** 9-month treatment. **(C)** Rotarod Test: 6-month Motor learning ability was calculated by Trial 9-Trial 1 latency to fall off the rotarod. MWM: Spatial learning ability was recorded during day1 to day5 training after **(D)** 6-month and **(I)** 9-month treatment. Heatmap represents the swimming route after **(E)** 6-month, and **(J)** 9-month treatment (representative images selected based on the group median value to best reflect central tendency). Mean escape latency after **(F)** 6-month, and **(I)** 9-month treatment, distance moved after **(G)** 6-month, and **(L)** 9-month treatment, and **(H)(M)** swimming velocity to the platform were recorded. Control, n=8, low-dose, n=9, high-dose, n=8. Normality was assessed using the Shapiro-Wilk test. Statistical differences among groups were analyzed by One-Way ANOVA, followed by Dunnett’s multiple comparison test. For datasets that failed normality, non-parametric Kruskal-Wallis was employed followed by Dunnett’s multiple comparison test. *p<0.05, **p<0.01, ***p<0.001 were considered statistically significant. Data are expressed as mean ± SD.

To investigate the chronic effect of SCFA treatment, cognitive performance was assessed at 6- and 9-months following treatment initiation. Both low-dose and high-dose SCFA groups exhibited significantly higher recognition indices in the NORT compared to controls at both 6 months (low-dose: p=0.0012 and high-dose: p=0.0001) and 9 months (low-dose: p=0.0016 and high-dose: p=0.001) (Fig. 2A and 2B). Learning ability was further evaluated using the rotarod test, which demonstrated that 6-month high-dose SCFA treatment significantly enhanced motor learning ability (p=0.0493; Fig. 2C). Similarly, in the MWM test, both SCFA-treated groups showed significantly reduced escape latencies (low-dose: p=0.0057 and high-dose: p=0.0021) (Fig. 2D, F, J and K), accompanied by shorter path lengths to reach the platform (Fig. 2E and J). Consistent with earlier observations, no significant differences in swimming speed were detected among groups (Fig. 2H and M). These findings suggest that long-term SCFA treatment can mitigate age-related cognitive decline.

### Long-term SCFAs prevented hippocampus atrophy and ventricular dilatation

**Figure 3.**
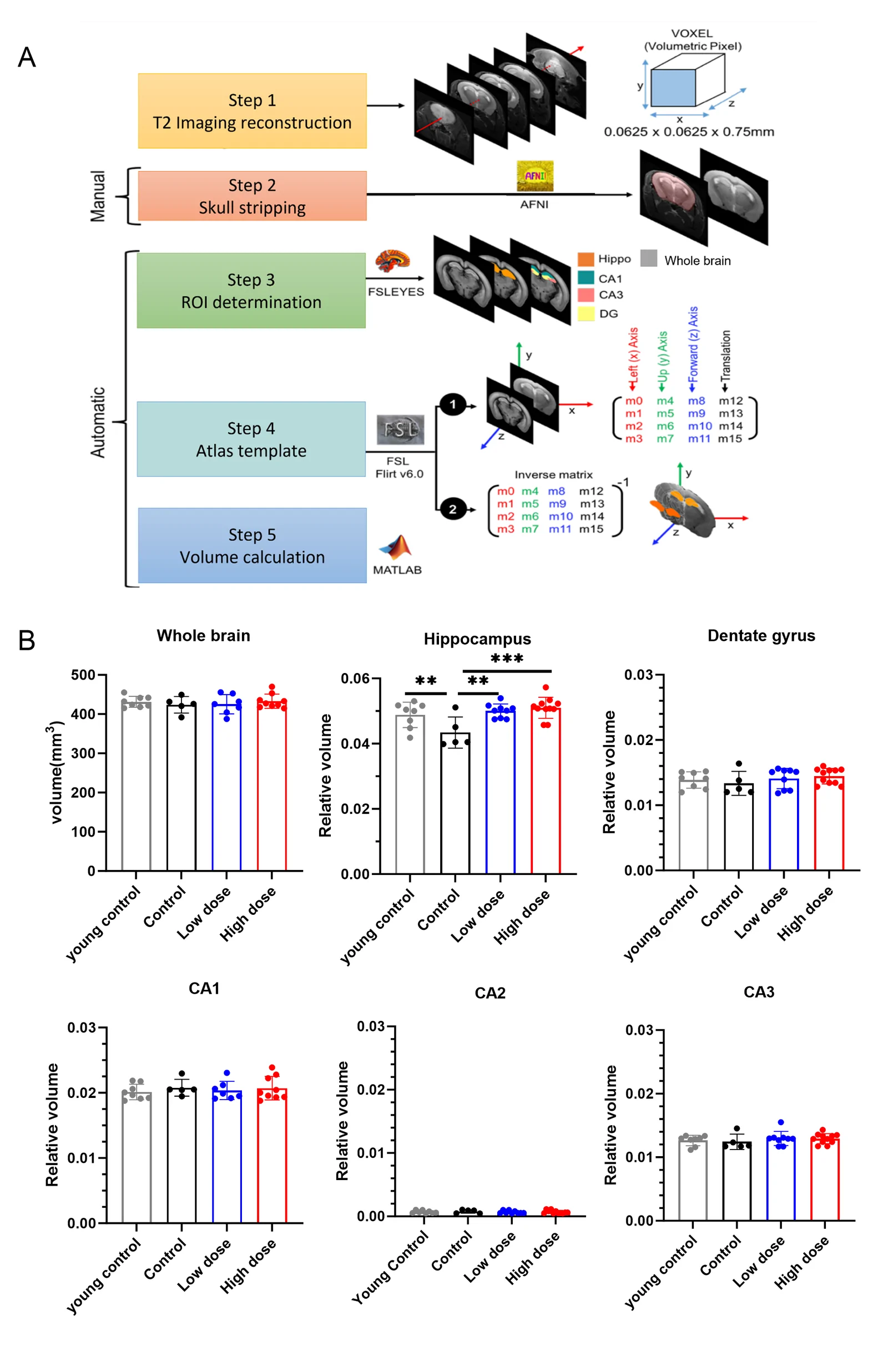
**SCFA treatment slows down hippocampus atrophy. (A)** Analysis procedure of 7T MRI, 12-month-old mice volume of **(B)** Whole brain, relative volume of (C) Hippocampus (D) DG (E) CA1 (F) CA2 (G) CA3. Young control, n=8, control, n=5, low-dose, n=9, high-dose, n=11. Normality was assessed using the Shapiro-Wilk test. Statistical differences among groups were analyzed by One-Way ANOVA, followed by Dunnett’s multiple comparison test. For datasets that failed normality, non-parametric Kruskal-Wallis was employed followed by Dunnett’s multiple comparison test. *p<0.05, **p<0.01, ***p<0.001 were considered statistically significant. Data are expressed as mean ± SD.

Age-associated cognitive decline is closely linked to structural brain changes, including hippocampal atrophy and ventricular dilatation [45, 46]. To explore the neuroanatomical effects of SCFAs, 7T MRI was used to quantify brain region volumes. An additional young control group (3-month-old mice) was included for comparison alongside 12-month-old untreated control, low-dose and high-dose SCFA treated mice. Figure 3A depicts the semi-automatic analysis of 7T MRI imaging. MRI images were preprocessed using MRIcroGL and converted to NifTi format, with an axis orientation and a voxel size of 0.0625×0.0625×0.75 mm. Skull stripping and segmentation were performed using AFNI, and regional volumes including the hippocampus, CA1, CA2, CA3, DG, and total brain volume were measured using FSLeyes.

**Figure 4.**
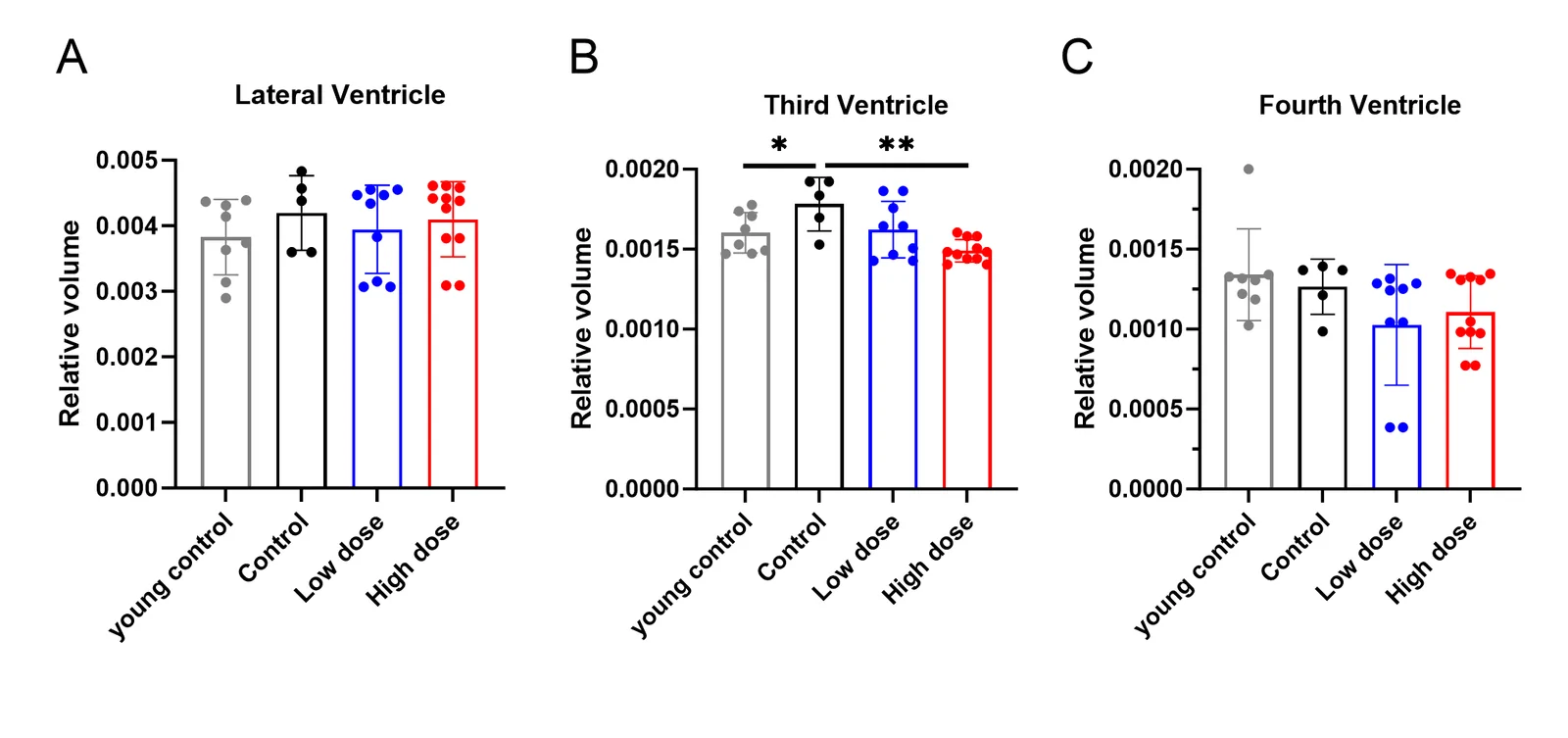
**SCFA treatment slows down dilation of ventricle.** 12-month-old mice relative volume of **(A)** Lateral Ventricle **(B)** 3rd Ventricle, and **(C)** 4th Ventricle. Young control, n=8, control, n=5, low-dose, n=9, high-dose, n=11. Normality was assessed using the Shapiro-Wilk test. Statistical differences among groups were analyzed by One-Way ANOVA, followed by Dunnett’s multiple comparison test. *p<0.05, **p<0.01. Data are expressed as mean ± SD.

Template alignment and volumetric calculations were completed using Matlab_R2018b. Total brain volume did not differ significantly among groups (Fig. 3B). The relative volume of each brain region was calculated as the ratio of the absolute volume of that specific brain region to the total whole-brain volume of the same subject, allowing for normalization across individuals with different brain sizes. As expected, aged control mice exhibited a significant reduction in hippocampal volume compared to young mice (p=0.0097). However, both low- (p=0.0029) and high-dose (p=0.0006) SCFA treatment effectively prevented hippocampal volume loss. The relative hippocampal volume demonstrated large effect sizes when compared to the aged control group (low-dose vs. control: d = 1.98; high-dose vs. control: d = 2.03). Subfield analysis revealed a trend toward preservation of dentate gyrus (DG) and CA3 subregions in SCFA-treated groups, though these differences did not reach statistical significance (DG, p=0.5272 and CA3, p=0.7146). Hippocampal subregions such as CA1 and CA2 exhibited only small or negligible differences (d < 0.6). Notably, the high-dose SCFA group, like young mice, displayed a significantly reduced 3rd ventricle volume compared to aged controls (Fig. 4B). The third ventricle (V3) volume was markedly reduced in the high-dose SCFA group, with a large negative effect size (*d* = -2.50), suggesting that SCFA may mitigate age-related ventricular enlargement. These effect size calculations reinforce the biological relevance of the volumetric changes beyond the reported p-values (Supplementary Table 1). These findings suggest that SCFA treatment may protect against aging-related hippocampal atrophy and ventricular dilation.

### The alteration in gut microbiota after long-term SCFAs correlated to hippocampal volume

**Figure 5.**
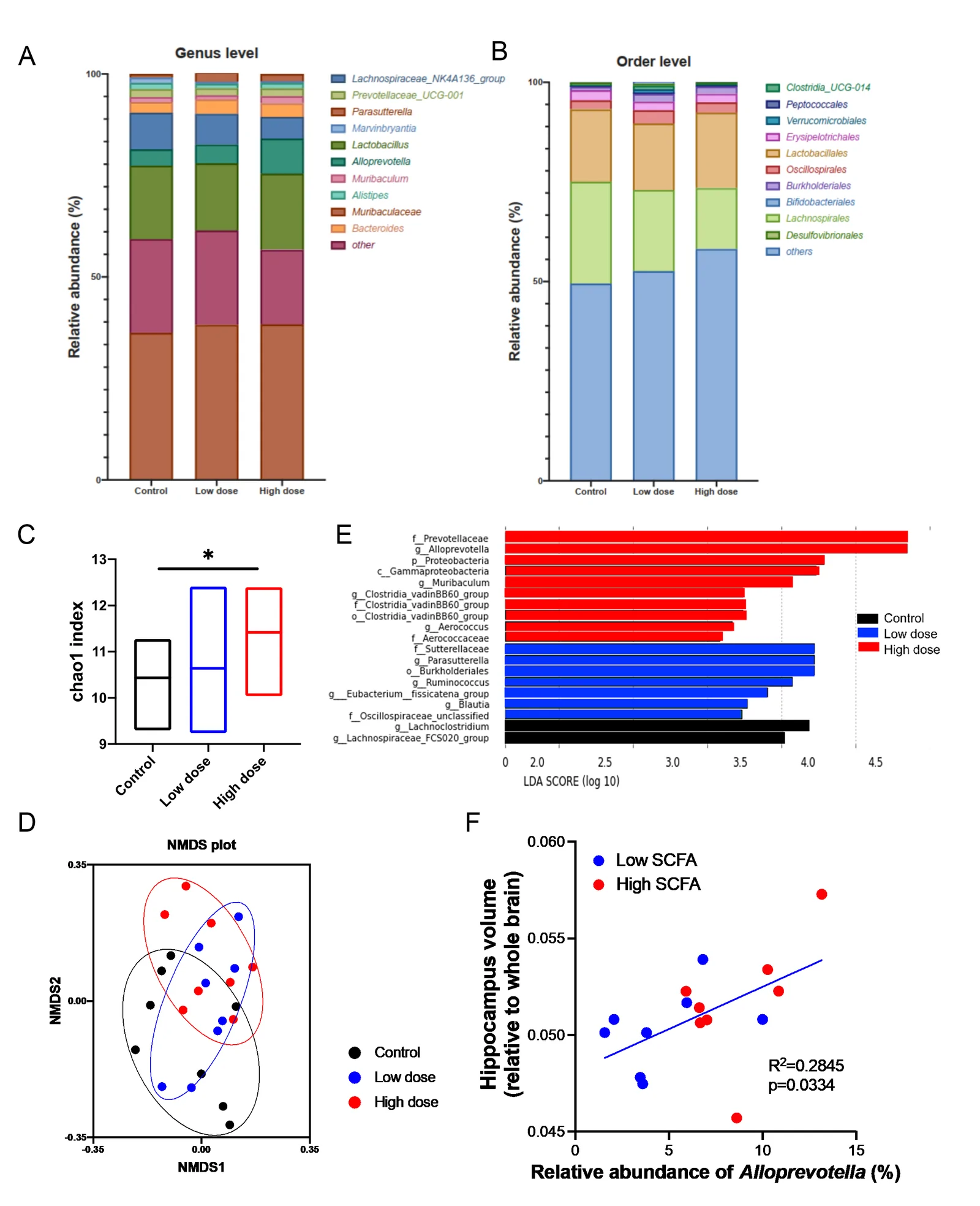
**SCFA treatment modulates gut microbial composition.** Gut microbiota signatures were expressed in terms of relative abundance of bacterial taxa at **(A)** genus and **(B)** order levels; **(C)** Alpha-diversity (Chao1 index), **(D)** Beta-diversity represented by non-metric multidimensional scaling (NMDS) plots based on UniFrac distances, and **(E)** Histograms of bacterial taxa across the three groups (cutoff was the linear discriminant analysis (LDA)>4 and p-value<0.05) p: phylum; c: class; o: order; f: family; g: genus; s: species. (F) Correlation between hippocampus volume and different dose of SCFAs. Control (n = 8), low-dose (n = 8), and high-dose (n = 8). Normality was assessed using the Shapiro-Wilk test. Statistical analysis was performed using one-way ANOVA. *p < 0.05, **p < 0.01 were considered statistically significant.

**Figure 6.**
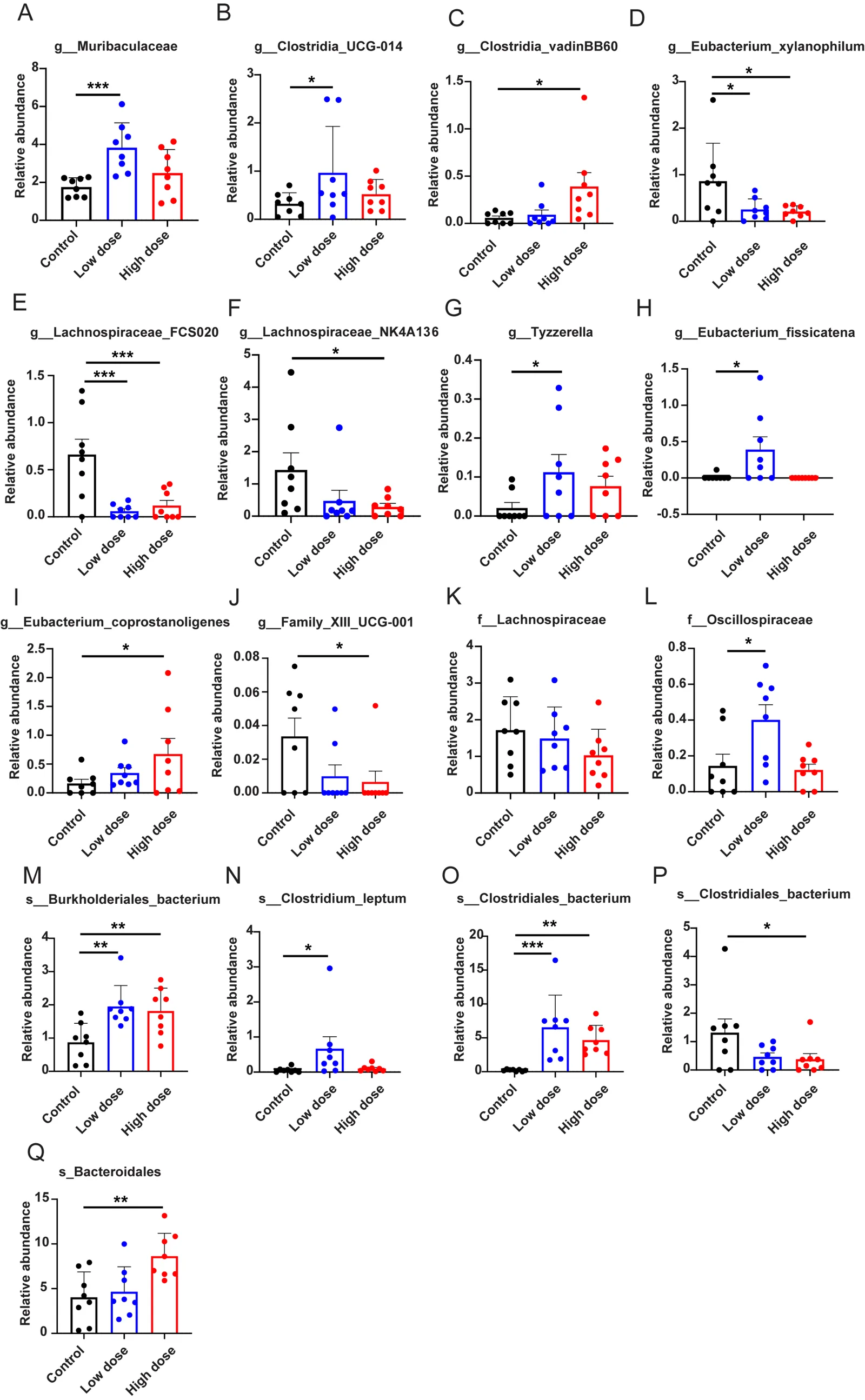
**Gut microbiome composition after 9-month SCFA treatment.** Gut microbiota signatures were expressed in terms of relative abundance of bacterial taxa at **(A-J)** genus **(K)(L)** family, and **(M-Q)** species levels. The relative abundance of bacteria across the three different groups. Black, blue, and red indicate bacteria with a higher abundance in the control (n = 8), low-dose (n = 8), and high-dose (n = 8). Normality was assessed using the Shapiro-Wilk test. Statistical analysis was performed using one-way ANOVA. *p < 0.05, **p < 0.01 were considered statistically significant.

To further explore whether gut microbiota alteration is associated with the mitigation of hippocampal changes by SCFAs. Fecal samples were collected after 9 months of treatment and analyzed by 16S rRNA sequencing. Taxonomic profiling revealed distinct shifts in microbiota composition at both the order and genus levels (Fig. 5A and B). The most abundant genre included *Bacteroides*, *Muribaculaceae, Alostipes, Murbaculum, Alloprevotella, Lactobacliilus, Marvinbryantia, Parasutterella, Prevo-tellaceae_UCG-001, Lachnosipiraceae_NK4A136_ group*. At the order level, we found that SCFA increased the relative abundance of *Bifidobacterium* (p=0.004), while reducing *Lachnospiraceae* (p=0.0016). Alpha diversity analysis showed that the high-dose SCFA group had a significantly higher Chao1 index compared to controls (p<0.05, Fig. 5C). Beta diversity analysis using NMDS ordination of UniFrac distances revealed clear clustering of gut microbial communities according to treatment (Fig. 5D). LEfSe analysis identified specific taxa enriched by SCFA treatment (Fig. 5E). Notably, *Alloprevotella* abundance was significantly elevated in the high-dose SCFA group, while *Parasutterella* was enriched in the low-dose group. Moreover, a positive correlation was observed between *Alloprevotella* abundance and hippocampal volume (p = 0.033; Fig. 5F), suggesting a potential link between microbiota composition and neuroprotection. Further analysis revealed that low-dose SCFA treatment increased the abundance of taxa such as *f_Oscillospiraceae*, *g_ Eubactreium_fissicatena, g_Clostrida_UCG-014, g_ Muribaculaceae, g_Tyzzerella, s_Clostridium_leptum, s_ Burkholderiales_bacterium, s_Clostridales_bacterium*, while the reduced taxa were *g_Lachnospiraceae_FCS20, g_Eubacterium_xylanophilum, g_Muribaculaceae*, compared to control. Additionally, high-dose SCFAs increased the relative abundance of *g_Eubacterium _coprostanoligene, s_Bacteroidales, g_Clostridia_ vadinBB60*, *s_Burkholderiales_bacterium, s_Clostri-dales_bacterium,* while decreasing *g_Lachno-spiraceae_ FCS020, g_Eubacterium_xylanophilum, g_ Family_XII_ UCG-001, g_Lanospiraceae_NK4A136, s_ Clostridales_ bacterium*, compared to control (Fig. 6).

### SCFAs reduced serum levels of TNF-α and IL-6, and suppressed hippocampal TLR4 expression

**Figure 7.**
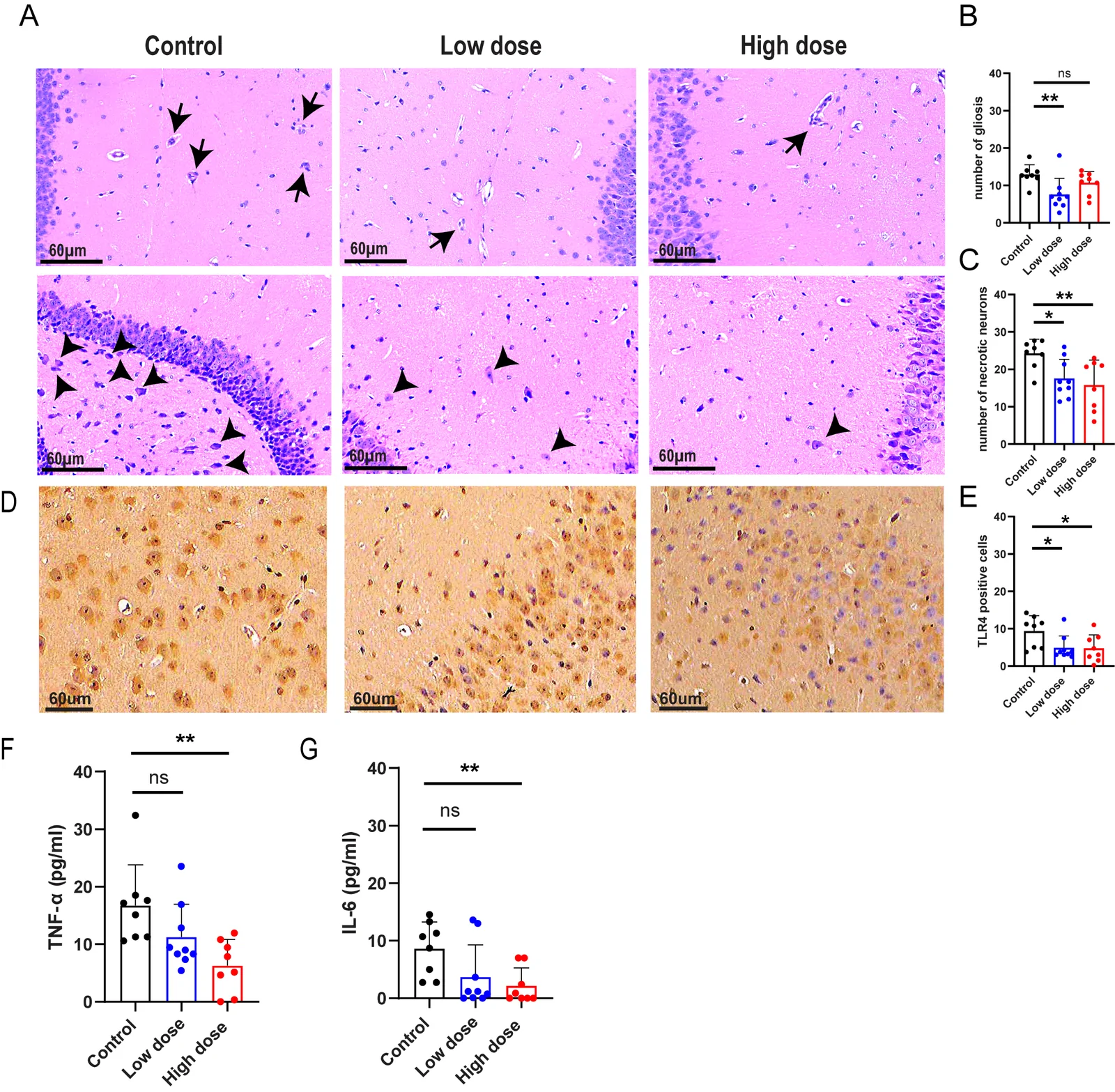
**Effects of SCFAs on inflammation in aged mice. (A)** Representative H&E-stained hippocampal sections show gliosis (indicated by black arrows in the upper panel) and neuronal necrosis (marked by black arrowheads in the lower panel) in mice with and without SCFA treatment. **(B. C)** Quantification of gliosis and necrotic neurons in control (n = 8), low-dose SCFA (n = 9), and high-dose SCFA (n = 8) groups. **(D)** Immunohistochemical staining illustrates TLR4-expressing cells in the hippocampus. **(E)** The absolute number of TLR4⁺ cells in the hippocampus across groups: control (n = 8), low-dose (n = 9), and high-dose (n = 8). (**F, G**) Serum levels of pro-inflammatory cytokines TNF-α and IL-6 were measured by ELISA test in control (n = 8), low-dose (n = 9), and high-dose (n = 8). Normality was assessed using the Shapiro-Wilk test. Statistical analysis was performed using one-way ANOVA, followed by Dunnett’s multiple comparison test. *p < 0.05, **p < 0.01 were considered statistically significant.

To determine whether SCFA treatment modulates inflammatory status, we assessed systemic and hippocampal markers of inflammation following chronic exposure. In the hippocampal region, histopathological examination using H&E staining revealed a significant reduction in necrotic neuronal profiles (low-dose, p = 0.0323; high-dose, p = 0.0087) and attenuated gliosis (low-dose, p = 0.0084) in SCFA-treated mice compared to controls (Fig. 7A–C). In addition, hippocampal expression of Toll-like receptor 4 (TLR4), a sentinel of innate immunity that orchestrates the transcriptional activation of cytokines including TNF-α and IL-6 during persistent neuroinflammation [47], was substantially reduced in SCFA-treated mice. Immunohistochemical analysis revealed a significant decline in TLR4-positive cells in the hippocampus of both low-dose (p = 0.0363) and high-dose (p = 0.0370) treatment groups (Fig. 7D and E). Circulating concentrations of the pro-inflammatory cytokines TNF-α and IL-6, commonly elevated during aging and implicated in the pathogenesis of neurodegenerative disorders [48], were notably attenuated in SCFA-treated groups. In particular, TNF-α and IL-6 levels were significantly diminished in the high-dose group (p = 0.006 and p = 0.003, respectively), relative to age-matched controls (Fig. 7F and G). These data imply that SCFAs may attenuate systemic inflammatory tone, a known contributor to age-related cognitive decline. Together, these results suggest that SCFAs might confer neuroprotection, at least in part, by dampening both systemic and central inflammatory cascades.

## DISCUSSION

SCFAs, produced from fermentation of dietary fiber by gut microbiota, have been shown to improve clinical outcomes of neuropsychiatric disorders [33]. However, its effect on aging remains unclear. In this study, we demonstrate that SCFAs significantly ameliorate age-related cognitive deficits, reduce hippocampal atrophy and ventricular dilation, and alter gut microbiota composition in aged mice. These improvements are accompanied by reduced neuroinflammation and downregulation of hippocampal Toll-like receptor 4 (TLR4), suggesting a link between SCFAs, microbiota modulation, and immune regulation in brain aging.

Our behavioral tests, including the novel object recognition test (NORT) and Morris water maze (MWM), revealed that both low- and high-dose SCFA treatment improved memory performance in aged mice. These results are consistent with prior studies demonstrating cognitive improvement following SCFA administration. For example, acetate gavage for four weeks improved cognitive deficits in an APP/PS1 Alzheimer’s model [49], while an SCFA mixture (67.5 mM acetate, 40 mM butyrate, 25.9 mM propionate) reduced stress in mice, although its effect on recognition memory was limited [50]. These findings underscore SCFAs’ potential in cognitive support, though degree of efficacy may depend on dose, duration, and disease context.

Neuroinflammation is a hallmark of aging and a key factor in neurodegeneration. We observed reduced levels of pro-inflammatory cytokines (TNF-α and IL-6) and downregulation of hippocampal TLR4 after SCFA treatment. TLR4 plays a crucial role in microglial activation and the production of inflammatory mediators in the aging brain [42, 51]. Its suppression suggests a mechanism by which SCFAs may alleviate neuroinflammation and cognitive decline. Supporting evidence includes prior studies showing that sodium butyrate reduces microglial activation and brain-penetrating cytokines [52–56], while acetate and butyrate attenuate TNF-α and IL-6 expression in vitro [57, 58]. However, SCFAs’ effects appear context dependent. For instance, SCFAs exacerbated neuroinflammation in a Parkinson’s disease mouse model [59], indicating that their immunomodulatory role varies depending on disease state and immune environment.

SCFAs also play a role in regulating microglial maturation and function [60, 61]. Butyrate, a known histone deacetylase inhibitor, has demonstrated neuroprotective effects, while acetate reduces IL-6 and TNF-α production in microglia [47]. These data support the notion that SCFAs modulate central immune responses and may exert neuroprotective effects via suppression of TLR4 signaling. The observed downregulation of hippocampal TLR4 following SCFA treatment suggests a mechanistic link between SCFAs and reduced neuroinflammation. Nonetheless, the precise role of TLR4 in mediating these neuroprotective effects requires further clarification. Future studies using TLR4-deficient mice or specific TLR4 inhibitors will be essential to determine whether SCFA-mediated TLR4 suppression is necessary for the observed benefits.

Our findings also underscore the role of gut microbiota in brain aging. SCFA treatment increased the relative abundance of *Clostridiales bacterium*, *Intestinimonas*, *Clostridium leptum*, and *Clostridia-UCG-01* microbes involved in butyrate production and tryptophan metabolism [62, 63]. High-dose SCFAs also increased levels of *Candidatus Arthromitus* and *Clostridium sp.*, taxa associated with enhanced SCFA production and immune homeostasis [64, 65]. Moreover, microbial phyla elicit distinct immune responses: *Bacteroidetes* and *Proteobacteria* stimulate IL-6, TNF-α, and IL-10, while *Proteobacteria* also promote IL-23 and IL-1β production via TLR activation [66]. Importantly, *Alloprevotella* abundance was significantly increased by SCFA treatment and positively correlated with hippocampal volume. A previous study by Sun *et al.* [67] reported that *Alloprevotella* in 3xTg-AD mice, which was restored after fecal microbiota transplantation (FMT), supporting its role in maintaining brain structure. Given that our findings are correlative, we suggest that SCFAs may help preserve hippocampal volume through microbiota modulation.

The gut-brain axis, a bidirectional communication network involving neural, immune, and metabolic signalling, is increasingly recognized as central to brain health [29]. SCFAs modulate this axis through regulatory effects on immune cells, including regulatory T cells (Tregs), and influence inflammation both locally and systemically [60]. Age-related dysbiosis has been shown to increase intestinal permeability and activate the hippocampal TLR4/NF-κB pathway, contributing to cognitive decline [42]. SCFA treatment may counteract this inflammatory cascade by restoring microbial balance and reducing TLR4 expression. Multiple studies highlight the cognitive relevance of gut microbiota composition. FMT from Alzheimer's disease patients induces cognitive dysfunction in mice [68–70], while SCFA-producing bacteria have been associated with greater hippocampal and white matter volumes [71]. Additionally, higher abundance of *Odoribacter* is associated with increased hippocampal and white matter volumes and reduced cerebrospinal fluid (CSF) volume [72]. Epidemiological studies further support a link between diet, microbiota, and cognition: higher dietary fiber intake is associated with better cognitive performance, including in older adults with chronic kidney disease [56]. These data reinforce the view that SCFAs contribute to improved memory, likely via microbiota-mediated pathways [73]. Although the observed correlation between *Alloprevotella* abundance and hippocampal volume is compelling, further studies employing functional approaches, such as germ-free mouse models or fecal microbiota transplantation will be necessary to confirm whether specific microbiota changes directly contribute to the observed neuroanatomical and cognitive improvements.

While our study demonstrates the causal role of SCFAs in mitigating age-related cognitive decline. Despite these promising findings, this study has limitations. Only male mice were used in this study to reduce variability due to the estrous cycle, a common consideration in preclinical neuroscience studies, which limits the generalizability of our findings. However, future studies should include both sexes to assess whether these findings extend to females and to better understand sex-dependent differences in gut-brain interactions.

In summary, SCFA treatment improved cognitive function, reduced hippocampal atrophy and ventricle enlargement, and modulated gut microbiota in aged mice. These benefits were associated with increased abundance of beneficial bacteria, particularly butyrate producers and those involved in tryptophan metabolism. SCFAs also reduced systemic inflammation and hippocampal TLR4 expression, suggesting their role in modulating age-related neuroinflammation. Our findings support the potential of SCFAs or dietary fiber as therapeutic strategies to combat cognitive aging and warrant further investigation in both preclinical and clinical settings.

## Supplementary Materials

The Supplementary data can be found online at: www.aginganddisease.org/EN/10.14336/AD.2025.0428.

## Data Availability

The original contributions presented in the study are included in the article and Supplementary Material, further inquiries can be directed to the corresponding authors.
