# Supplementary material for: Short-Chain Fatty Acids Improve Hippocampal Atrophy, Ventricular Dilatation and Cognitive Function Decline in Aged Mice
Source: Aging Dis. 2025 May 26;17(3):1737–55. doi: 10.14336/AD.2025.0426 (PMC13061538; doi:10.14336/AD.2025.0426)
Supplement: Supplementary file 1 — The Supplementary data can be found online at: www.aginganddisease.org/EN/10.14336/AD.2025.0428. [file AD-17-3-1737-s.pdf]

SUPPLEMENTARY DATA

# Short-Chain Fatty Acids Improve Hippocampal Atrophy, Ventricular Dilatation and Cognitive Function Decline in Aged Mice

**Pei-Ju Lee, Yu-Chun Lo, You-Yin Chen, Chaur-Jong Hu, Yen-Kuang Lin, Quoc Thao Trang Pham, Nicholas Keyi Sim, Chee Kin Then, Shing-Chuan Shen**

© 2025. Lee PJ et al. Published online at http://www.aginganddisease.org/EN/10.14336/AD.2025.0426

# SUPPLEMENTARY DATA

## SUPPLEMENTARY TABLE

**Supplementary Table 1. Relative brain region volumes (mean ± SD) and effect sizes (Cohen's *d*) comparing SCFA-treated and control groups.** Relative volumes of brain structures (mean ± SD) and Cohen's d effect sizes for comparisons between aged control mice and SCFA-treated groups. Effect sizes were calculated to reflect the magnitude of difference independent of sample size: values of 0.2, 0.5, and 0.8 were interpreted as small, medium, and large effects, respectively. Hippocampus and subregion volumes were normalized to whole brain volume to control individual variability in brain size. SCFA treatment (both low and high dose) was associated with a substantial effect size in preserving hippocampal volume compared to untreated aged mice. control, n=8, low dose, n=9, high dose, n=8.

| | Young Control | Aged Control | low-dose SCFAs | high-dose SCFAs | low vs control (*d*) | high vs control (*d*) |
|---|---|---|---|---|---|---|
| Whole brain ($mm^3$) | 431.21 ± 13.07 | 423.83 ± 18.80 | 425.05 ± 22.76 | 432.90 ± 17.01 | 0.07 | 0.52 |
| Relative Hippocampus | 0.0488 ± 0.0037 | 0.0434 ± 0.0043 | 0.0500 ± 0.0020 | 0.0510 ± 0.0031 | 1.83 | 2.01 |
| Relative CA1 | 0.0201 ± 0.0011 | 0.0208 ± 0.0012 | 0.0204 ± 0.0013 | 0.0207 ± 0.0017 | -0.032 | -0.04 |
| Relative CA2 | 0.00069 ± 0.00021 | 0.00076 ± 0.00025 | 0.00066 ± 0.00025 | 0.00069 ± 0.00024 | -0.4 | -0.19 |
| Relative CA3 | 0.0126 ± 0.00077 | 0.0124 ± 0.0011 | 0.0129 ± 0.00105 | 0.0129 ± 0.00076 | 0.54 | 0.52 |
| Relative DG | 0.01386 ± 0.00118 | 0.01335 ± 0.00165 | 0.01408 ± 0.00147 | 0.01445 ± 0.00113 | 0.5 | 0.81 |
| Lateral Ventricle | 0.03826 ± 0.00053 | 0.00420 ± 0.00051 | 0.00394 ± 0.00063 | 0.00409 ± 0.00055 | 0.47 | 0.78 |
| Third Ventricle | 0.00160 ± 0.00012 | 0.00178 ± 0.00015 | 0.00162 ± 0.00017 | 0.00149 ± 0.00007 | -1.11 | -2.24 |
| Fourth Ventricle | 0.00134 ± 0.00027 | 0.00127 ± 0.00015 | 0.00103 ± 0.00036 | 0.00111 ± 0.00022 | -0.86 | -0.5 |

© 2025. Lee PJ et al. Published online at http://www.aginganddisease.org/EN/10.14336/AD.2025.0426

# SUPPLEMENTARY DATA

## SUPPLEMENTARY FIGURE

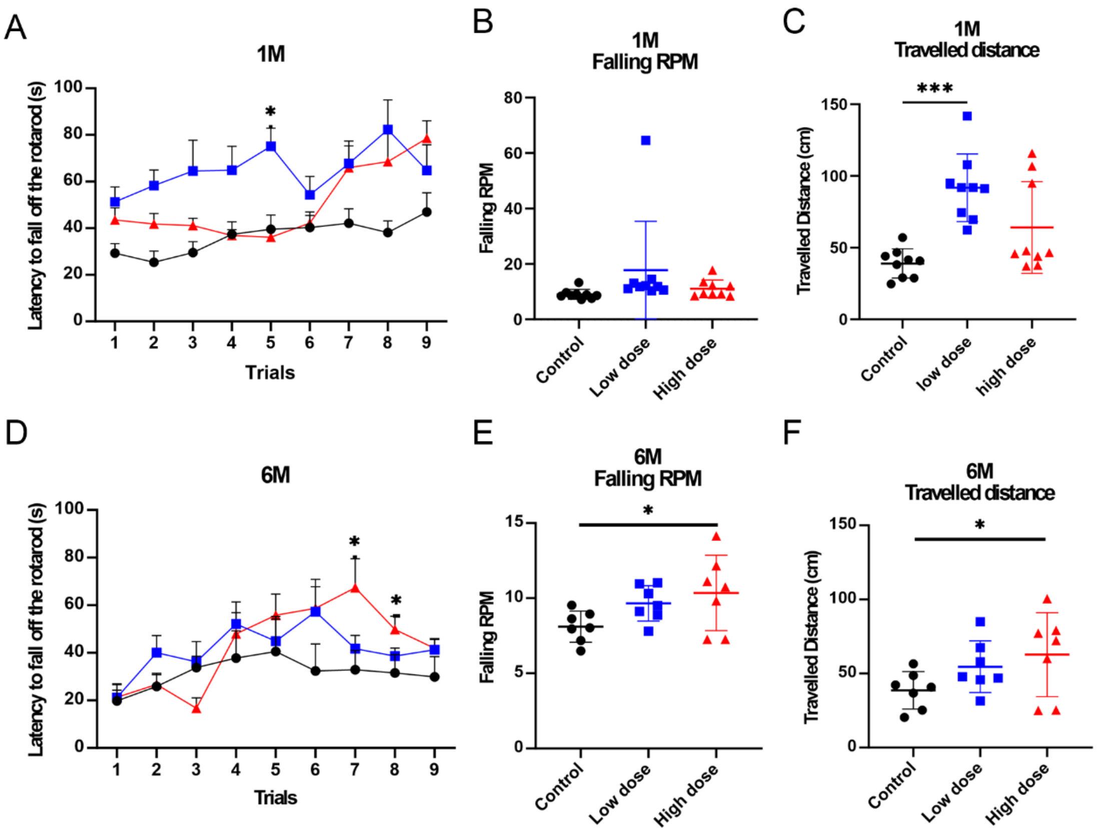


**Supplementary Figure 1. Assessment of motor learning ability after Short-term and Long-term SCFAs using Rotarod test.** Latency to fall off the rotarod was recorded during 9 trials after **(A)** 1-month and **(D)** 6-month treatment; **(B)(E)** RPM when mice fall off the rotarod, and **(C)(F)** Travel distance was recorded. Control, n=8, Low dose, n=9, High dose, n=8. We confirm that normality was assessed using the Shapiro-Wilk test. Statistical analysis was performed using one-way ANOVA, followed by Dunnett's multiple comparison test. *$p < 0.05$, **$p < 0.01$, ***$p<0.001$, were considered statistically significant. Data are expressed as mean ± SD.

© 2025. Lee PJ et al. Published online at http://www.aginganddisease.org/EN/10.14336/AD.2025.0426

# SUPPLEMENTARY DATA

SUPPLEMENTARY DATA

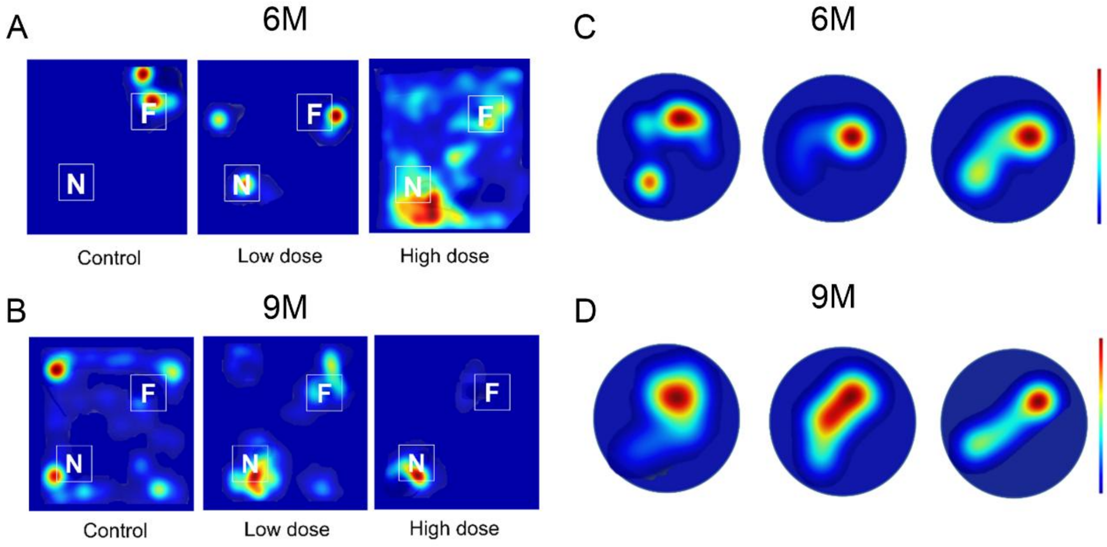


**Supplementary Figure 2. Assessment of short-term memory and spatial memory after long-term SCFAs using NORT and MWM test.** The heatmap shows the movement of mice around the familiar and novel objects after **(A)** 6 months and **(B)** 9 months of treatment. The heatmap represents the swimming routes in the MWM test after **(C)** 6 months and **(D)** 9 months of treatment.

© 2025. Lee PJ et al. Published online at http://www.aginganddisease.org/EN/10.14336/AD.2025.0426

# SUPPLEMENTARY DATA

SUPPLEMENTARY DATA

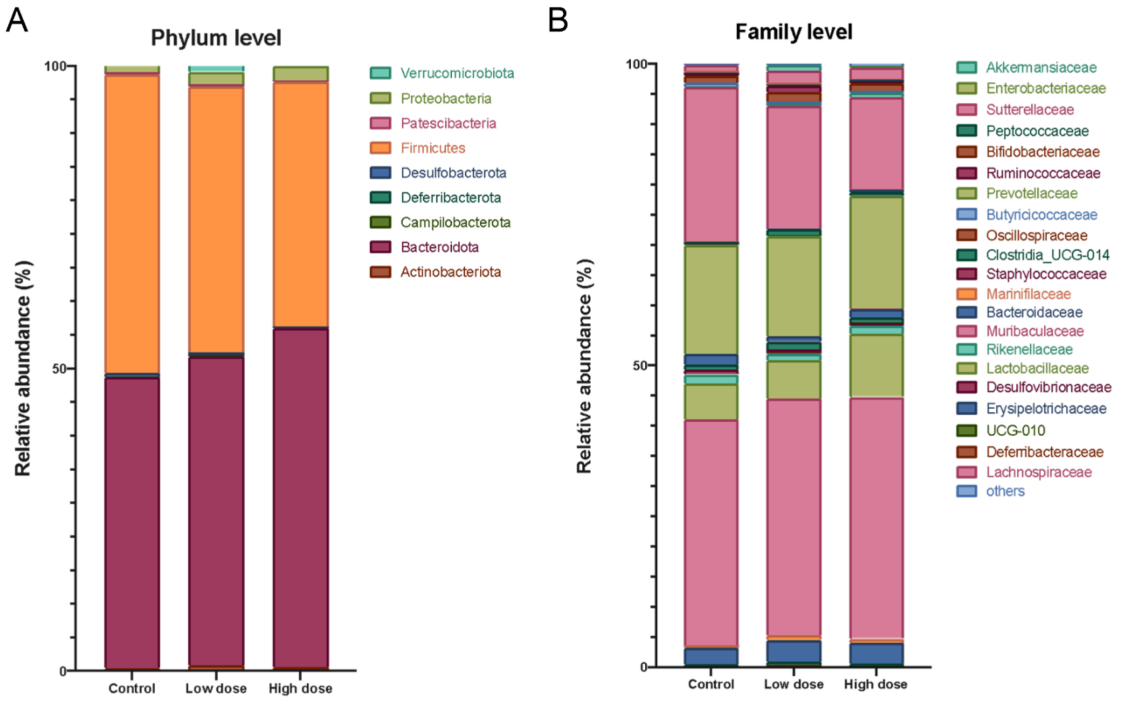


**Supplementary Figure 3. Different doses of SCFAs change gut microbiome composition.** Relative abundance of the major taxa at **(A)** phylum and **(B)** family level. Black, blue, and red indicate bacteria with a higher abundance in the control (n = 8), low-dose (n = 8), and high-dose (n = 8), *p<0.05, **p<0.01, ***p<0.001. Data are expressed as means ± SD.

© 2025. Lee PJ et al. Published online at http://www.aginganddisease.org/EN/10.14336/AD.2025.0426
